# Observation of T_2_-like coherent optical phonons in epitaxial Ge_2_Sb_2_Te_5_/GaSb(001) films

**DOI:** 10.1038/srep02965

**Published:** 2013-10-16

**Authors:** A. Shalini, Y. Liu, U.A.S. Al-Jarah, G. P. Srivastava, C. D. Wright, F. Katmis, W. Braun, R. J. Hicken

**Affiliations:** 1 School of Physics and Astronomy, University of Exeter, Stocker Road, Exeter, EX4 4QL, UK; 2 School of Engineering, Computing and Mathematics, University of Exeter, North Park Road, Exeter, EX4 4QF, UK; 3 Paul Drude Institute for Solid State Electronics, Hausvogteiplatz 5-7, 10117 Berlin, Germany; 4 Createc Fischer & Co. GmbH, Industriestr. 9, 74391 Erligheim, Germany

## Abstract

The phonon spectrum of Ge_2_Sb_2_Te_5_ is a signature of its crystallographic structure and underlies the phase transition process used in memory applications. Epitaxial materials allow coherent optical phonons to be studied in femtosecond anisotropic reflectance measurements. A dominant phonon mode with frequency of 3.4 THz has been observed in epitaxial Ge_2_Sb_2_Te_5_ grown on GaSb(001). The dependence of signal strength upon pump and probe polarization is described by a theory of transient stimulated Raman scattering that accounts for the symmetry of the crystallographic structure through use of the Raman tensor. The 3.4 THz mode has the character of the 3 dimensional T_2_ mode expected for the O_h_ point group, confirming that the underlying crystallographic structure is cubic. New modes are observed in both Ge_2_Sb_2_Te_5_ and GaSb after application of large pump fluences, and are interpreted as 1 and 2 dimensional modes associated with segregation of Sb.

Alloys such as Ge_2_Sb_2_Te_5_ have attracted great interest due to their favorable properties for phase change memory applications^1^. The use of Ge_2_Sb_2_Te_5_ for optical disk coatings is well established while more recently Ge_2_Sb_2_Te_5_ has also been used in the development of phase change random access memory^2^ and within new paradigms for bio-inspired computing^3^,^4^. The popularity of Ge_2_Sb_2_Te_5_ stems from the fast and reversible phase transition that occurs between its amorphous and cubic crystalline phases, and the strong contrast in electrical conductance and optical reflectance that these phases exhibit. The cubic phase has a distorted rock-salt structure with a high density of vacancies. The distribution of vacancies and hence the bonding within the material, and its modification during the phase transition, remains poorly understood. Knowledge and understanding of the phonon spectrum can provide a fingerprint of the crystallographic structure while large amplitude phonons may appear as a precursor to the phase transition itself. Although several studies of coherent optical phonon (COP) dynamics have been performed upon both amorphous and polycrystalline Ge_2_Sb_2_Te_5_^5^,^6^,^7^,^8^,^9^, no such study has been conducted on a Ge_2_Sb_2_Te_5_ single crystal. Therefore the growth of epitaxial Ge_2_Sb_2_Te_5_ films upon GaSb(001) substrates^10^ by molecular beam epitaxy provides an opportunity to explore the character of the phonon spectrum of Ge_2_Sb_2_Te_5_.

Time-resolved optical pump-probe measurements can provide information about both the ultrafast relaxation of photo-excited carriers and the phase and amplitude of COPs^11^,^12^. By varying the polarization of the pump and probe beams relative to the crystallographic axes, the symmetry of a particular COP can be deduced, and insight can be gained into the non-thermal nature of phonon excitation and the structural phase transition at large fluence. In this article, we present time resolved reflectance (R) and anisotropic reflectance (AR) measurements of GaSb(001) and Ge_2_Sb_2_Te_5_/GaSb(001). The results are interpreted in terms of a theory of transient stimulated Raman scattering^11^ that can account for the excitation of phonons by either impulsive stimulated Raman scattering (ISRS), which can be considered to include displacive excitation of coherent phonons, or the action of an optically induced space charge (SC) field^13^. The dependence of the signals upon the pump and probe polarization is accounted for through use of the Raman tensor. The dominant mode observed in AR measurements of Ge_2_Sb_2_Te_5_ will be shown to be the T_2_-like mode of the cubic structure. New modes observed after the application of large pump fluences are interpreted as 1 and 2 dimensional modes of Sb, while a longer-lived transient AR signal is explained in terms of the piezoelectric response of the GaSb to a SC field.

## Results

**Figure 1:**
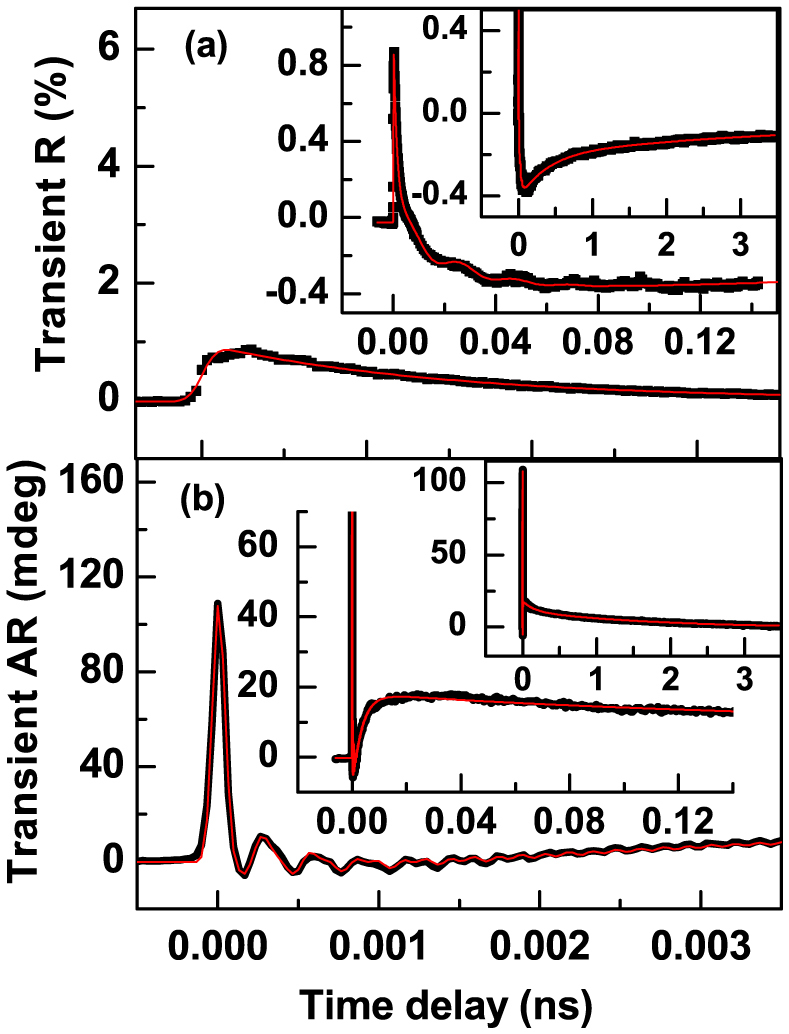
Typical time resolved (a) R and (b) AR signals for Ge_2_Sb_2_Te_5_/GaSb(001) with the pump and probe electric fields aligned parallel to the [110] and [100] axes respectively. The solid points represent the experimental data while the red/lines are the fitted curves described within the main text.

The transient reflectance (R) response of an epitaxial Ge_2_Sb_2_Te_5_(15 nm)/GaSb(001) structure, in which the [100] axes of the Ge_2_Sb_2_Te_5_ and GaSb are parallel is shown in Fig. 1(a) and is similar to that observed in GaSb(001) (shown in Supplementary Information S1). The red curve is a fit to a phenomenological fitting function. The procedure for fitting the R and AR scans is described within Supplementary Information S2. The signal is plotted on three different time scales so as to reveal all of its principal features. The initial rise of the signal occurs within about 200 fs and is associated with photoexcitation and thermalization of an electron-hole plasma. Weak oscillations with frequency of 4.2–4.5 THz are observed until about 1 ps time delay. The signal then changes sign after about 6 ps before reaching a minimum after about 70 ps. This region is well fitted by two exponential functions with relaxation times of 2.0 ± 0.1 ps and 13.1 ± 0.5 ps, which can be attributed to the interaction of the electrons with incoherent optical and acoustic phonons respectively^14^,^15^. Weak superimposed oscillations with frequency of 43 GHz are observed until about 150 ps and can be attributed to longitudinal coherent acoustic phonons propagating normal to the plane of the sample. The final relaxation towards the ambient state is well fitted by two exponential terms. The first has relaxation time of 0.42 ± 0.05 ns and can be assigned to carrier diffusion from the pumped spot. A longer time of 5.4 ± 0.5 ns can be associated with thermal conduction within the lattice. Similar relaxation behavior has been observed previously in Si^16^,^17^. The form of the transient R response was found to be independent of the pump and probe polarization except for a small variation in amplitude close to zero time delay. The similarity of the R signals obtained from the Ge_2_Sb_2_Te_5_/GaSb and GaSb samples suggests that the signals are dominated by the response of the homo-epitaxial GaSb layer and substrate.

The AR response of the Ge_2_Sb_2_Te_5_/GaSb(001) structure is shown in Fig. 1(b) for the pump and probe electric fields parallel to the [110] and [100] axes respectively. The signal contains a large peak centred at zero time delay that lies within the temporal overlap of the pump and probe pulses, two oscillatory components of 6.7 and 3.4 THz frequency superimposed on a multi-exponential background, and a slower (ns scale) relaxation. The initial peak arises from the specular optical Kerr effect^18^, an optically induced birefringence, and has maximum amplitude when the pump and probe electric fields lie 45° apart. The peak occurs because the pump modifies the electron momentum distribution within the surface of the sample and is short-lived due to the small momentum relaxation time^19^. The multi-exponential background is fitted well by the sum of three terms with relaxation times of 500 fs, 3.0 ps, and 137 ps. These terms may be associated with the momentum relaxation of charge carriers, and incoherent optical and acoustic phonons respectively^20^. The slowest relaxation time of 1.6 ns is associated with relaxation of strain in the GaSb that results from its piezolelectric response to a SC field. Oscillations of THz frequency have been widely observed in R measurements conducted on other materials^12^,^21^,^22^,^23^,^24^,^25^. If the fitted initial peak and multi-exponential background are subtracted from the AR data then the residual is well described by the sum of two damped sinusoids (*i* = 1, 2) of the form 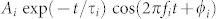, allowing the mode amplitudes *A_i_*, frequencies *f_i_*, relaxation times *τ_i_*, and phases *ϕ_i_*, to be determined.

AR measurements made upon the reference GaSb structure revealed largely similar features (shown in Supplementary Information S1). However the 3.4 THz mode was observed only in the Ge_2_Sb_2_Te_5_/GaSb structure, confirming that it originates in the Ge_2_Sb_2_Te_5_ layer. Similar relaxation times were obtained for the multi-exponential background and long relaxation terms, albeit with some difference in the amplitudes.

**Figure 2:**
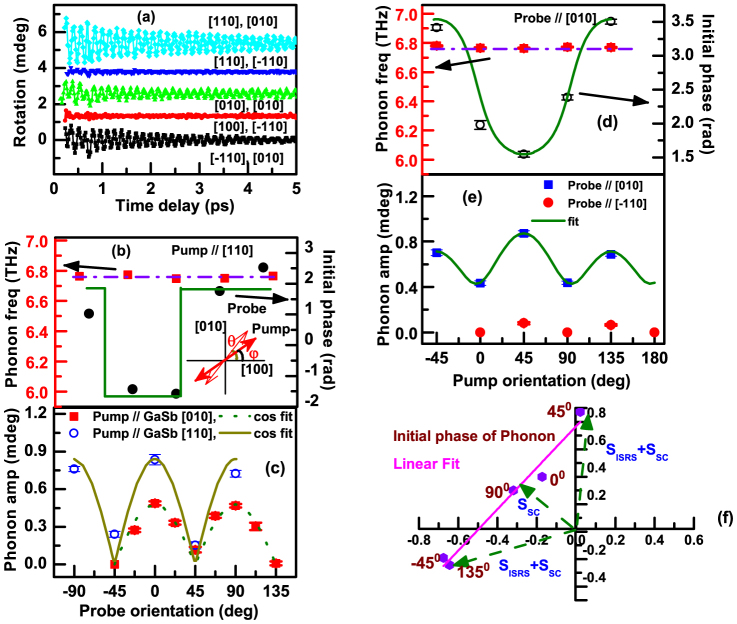
Dependence of frequency, amplitude and phase of phonon oscillations in GaSb upon the orientation of the pump and probe electric fields. (a) Typical oscillatory signals after subtraction of the initial peak and multi-exponential background (Labels represent the orientation of pump and probe polarization from GaSb[100] axis respectively). (b) Frequency and phase, and (c) amplitude, are plotted as the probe polarization is varied. (d) Frequency and phase, and (e) amplitude, are plotted as the pump polarization is varied. The angles *φ* and *θ* used to define the orientation of the pump and probe electric fields are shown within the inset to (b). (f) Data from (d) and (e) are plotted in the Argand plane. The purple straight line fit in (f) has been used to generate the continuous curves in (d) and (e).

Further measurements were made as the planes of polarization of the pump and probe beams were varied relative to the crystallographic axes of the sample. Figure 2 shows typical oscillatory components in panel (a), and the variation of their frequency, amplitude and phase in panels (b–e), for the GaSb structure. The variation of the amplitude of the ns transient in the AR response is presented in the Supplementary Information S3. The angles *φ* and *θ* used to define the orientation of the pump and probe electric fields relative to the [100] axis are shown within the inset to panel (b). From panels (b) and (d) the frequency is seen to have an essentially constant value of 6.77 THz. From panels (a) and (c) the oscillations are seen to almost vanish when the probe electric field lies along <110> and have maximum amplitude when it lies parallel to <100>. The variation of amplitude and phase in (c) and (b) respectively suggests an overall cos(2*θ*) dependence upon probe polarization. Panels (d) and (e) reveal a more complicated dependence upon the pump polarization. The amplitude is a minimum when the pump electric field lies parallel to <100> but has different values when it is parallel to [110] and [−110]. The range of phase of about 2 rad suggests contributions from different excitation mechanisms of different relative phase. However, when the amplitude and phase data of panels (e) and (d) respectively are plotted in the Argand plane, in panel (f), the data points are found to lie on a straight line.

## Discussion

GaSb has the zincblende structure with T_d_ point group symmetry and *A_1_*, *A_2_*, *E*, *T_1_* and *T_2_* irreducible representations at the zone centre^26^,^27^. Of the associated phonon modes only the *T_2_* mode is Raman active. At the center of the Brillouin zone, the three dimensional mode splits into a non-degenerate longitudinal optical (LO) phonon and doubly degenerate transverse optical (TO) phonons with slightly lower frequency^28^. Raman scattering studies^29^ have observed the LO phonon at 7.08 THz while neutron scattering^30^ measurements observe both LO and TO modes and suggest that the TO phonon is detected in the present experiments.

A theory of transient stimulated Raman scattering^11^ has been applied to the interpretation of the experimental results (see Supplementary Information S4). In this theory the amplitude of the COP excited by ISRS is proportional to a contraction of the relevant Raman tensor with the pump electric field. Then the probe beam is resolved into two orthogonally polarized components and the Raman tensor is contracted with each in turn. The transient R and AR signals are proportional to the sum and difference of these contractions respectively, and also to the phonon amplitude. Using the well-known form of the Raman tensor for the 5 irreducible representations, the theory predicts that only the *A*_1_, *A*_2_ and *E* COPs should be observed in the transient R measurements. For transient AR measurements only the T_2z_ mode with atomic displacement parallel to the [001] axis should appear, with amplitude proportional to cos(2*θ*)sin(2*φ*). A similar expression may be obtained from a simple model that considers the orientation of the pump and probe electric fields relative to inequivalent bonds within the zincblende structure (see Supplementary Information S5).

While the cos(2*θ*)sin(2*φ*) form describes observations of the T_2z_ COP in Ge(001)^23^, it does not fully account for the dependence on pump polarization observed in Fig. 2(d–f). Studies of COPs in GaAs^12^,^24^ have shown that the T_2z_ mode may also be excited by an optically induced SC field. Here photo-excited electrons and holes become spatially separated, either due to diffusion and because of their different mobilities in the photo-Dember effect^31^, or due to drift in opposite directions under the influence of the built-in electric field near an interface^13^. The latter mechanism seems more likely in the present case but both mechanisms generate an additional space charge field on sub-ps timescales. In a polar compound such as GaAs or GaSb, the oppositely charged basis ions experience an impulse in opposite directions normal to the plane of the film. This leads to an oscillatory motion of T_2z_ character superimposed upon an offset of the basis atom positions that gives rise to the long-lived transient within the AR response (see Supplementary Information S7). The amplitude of the T_2z_ COP excited by the SC field again exhibits a cos(2*θ*) dependence upon the probe polarization but is independent of the pump polarization.

ISRS and the SC field are expected to excite COPs of different phase. In ISRS the pump selectively excites bonds of different orientation, providing the driving force for the T_2z_ mode and leading to an electron-hole distribution that is anisotropic in k-space. While the bonding will be weakened on the timescale of the electron-hole recombination time (>100 ps), the electron and hole momentum distributions, and hence the inequivalent strength of bonds of different orientation, relax on fs timescales. The driving force for the T_2z_ phonon will therefore be very short-lived and “impulsive” in character. The rise of the SC field is limited by the drift of carriers within the built-in potential, and so the excitation of the COP will be delayed. The form of the data plotted in the Argand plane in Figure 2(f) can now be understood. When *φ* = 0 and 90°, the amplitude of the COP excited by ISRS is expected to vanish, revealing the amplitude and phase of excitation due to the SC field, which is represented by the vector **S**_SC_. As *φ* is varied, the amplitude of the phonon excited by ISRS varies, but with constant phase, tracing out a straight line that passes through the origin. The net effect of ISRS and SC field excitation is a straight line, offset from the origin. It is straightforward to fit a straight line to the data within the Argand plane, and hence generate the curves shown in Figure 2 (d–e) (see Supplementary Information S6).

**Figure 3:**
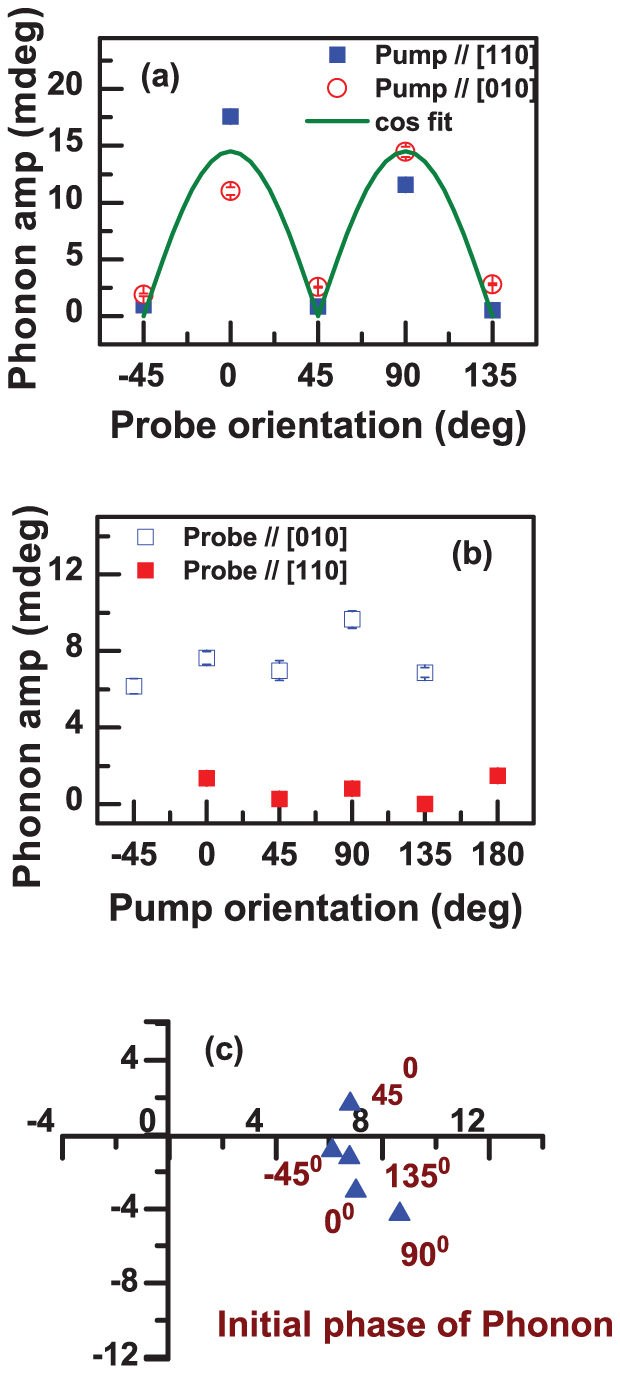
Dependence of the amplitude and phase of the 3.4 THz mode observed in Ge_2_Sb_2_Te_5_/GaSb(001) upon (a) probe and (b) pump polarization. (c) The dependence of amplitude and phase upon the pump polarization are plotted in the Argand plane.

The COPs observed in the transient AR response of Ge_2_Sb_2_Te_5_/GaSb(001) were analyzed in a similar manner. Fitting yielded two oscillatory components superimposed on a complicated background. The frequency and relaxation time of the first COP of larger amplitude were 3.4 ± 0.1 THz and 0.5 ps respectively, and 6.79 ± 0.02 THz and 3.0 ps respectively for the second COP. The frequencies were again found to be independent of the pump and probe polarization (see Supplementary Information S7). The 6.79 THz COP can again be attributed to the zone centre TO phonon of the GaSb homoepitaxial layer and substrate, while the 3.4 THz COP originates in the Ge_2_Sb_2_Te_5_ layer. The variation of the amplitude and phase of the 6.79 THz COP is consistent with that in Fig. 2 although less clear due to the smaller signal amplitude (see Supplementary Information S7). Fig. 3(a) and (b) show the dependence of the amplitude of the 3.4 THz COP upon the probe and pump polarization respectively. While the dependence upon probe polarization is similar to that observed in Fig. 2(c), there is only a weak dependence upon pump polarization. The data is plotted in the Argand plane in Figure 3(c). The clustering of data points close to the horizontal axis indicates that the phase of the COP is also insensitive to the pump polarization. Further plots of the phase of the 3.4 THz COP, and the amplitude of the 6.77 THz COP and the longer-lived transient in the AR signal are presented in the Supplementary Information S7.

The epitaxial Ge_2_Sb_2_Te_5_ film exhibits a distorted rock-salt structure with about 20% of the Ge/Sb atomic sites being vacant. The ideal rock-salt structure has O_h_ symmetry with five irreducible representations, usually denoted as *A*_1g_, *A*_2g_, *E_g_*, *T*_1g_ and *T*_2g_^26^ but none of the associated phonon modes are Raman active^26^,^27^. As for zincblende, the threefold T_2_ mode is split into a LO phonon and doubly degenerate TO phonons at the zone centre, the LO mode having slightly higher frequency^32^. The Raman tensor for the T_2_ mode has the same form for structures of both O_h_ and T_d_ symmetry. However, the structure of Ge_2_Sb_2_Te_5_ is only ‘rock salt like'. Ordering of vacancies and the displacement of ions from their positions in the ideal rock salt structure may remove the inversion symmetry, allowing 1^st^ order Raman scattering and also excitation of COPs by a SC field. Indeed the character of the allowed phonon modes may be modified. For example the zone-center T_2_ mode might be transformed into 1-D A and 2-D E modes if the symmetry of the crystal is reduced.

In previous studies of amorphous and polycrystalline Ge_2_Sb_2_Te_5_ films, a 3.4 THz mode observed in the R signal was assigned A_1g_ character^5^,^6^,^7^,^8^. However, Merlin's theory (described in Supplementary Information S4) predicts that the A and E modes should be observed only within the R signal, with no dependence on pump or probe polarization, while being absent from the AR signal. The weak oscillations at 4.2–4.5 THz in the R signal in Fig. 1(a) have frequency close to those observed previously^5^,^6^,^9^ and are attributed to an A mode. However, the observation of the 3.4 THz mode in the AR signal, and its dependence on probe polarization, point to T_2_-like character. Its frequency suggests that Sb-Te bonds play an important role^5^. In contrast to the 6.77 THz T_2z_ mode of GaSb, the amplitude and phase of the T_2_-like mode in Ge_2_Sb_2_Te_5_ have only a weak and unclear dependence on the pump polarization. This suggests that the COP is excited by a SC field, with negligible contribution from ISRS. Excitation may occur within the Ge_2_Sb_2_Te_5_ if broken inversion symmetry causes the Ge_2_Sb_2_Te_5_ to become piezoelectric. Alternatively the response of the GaSb to a SC field may generate impulsive excitation of the Ge_2_Sb_2_Te_5_ at the GaSb/Ge_2_Sb_2_Te_5_ interface.

**Figure 4:**
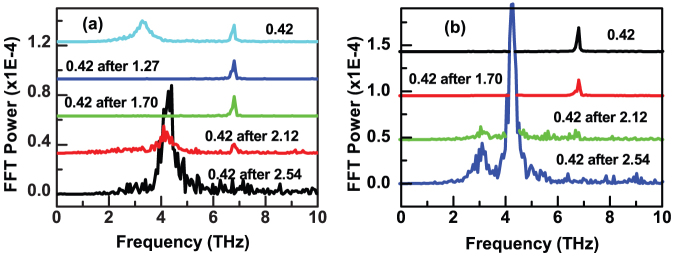
Power spectra obtained from AR signals measured with a pump fluence of 0.42 mJ/cm^2^, after exposure of as-deposited (a) Ge_2_Sb_2_Te_5_/GaSb and (b) GaSb samples to the higher pump fluences indicated in the plot.

Previous studies^31^,^33^,^34^,^35^ have shown that structural phase transitions can be induced in Ge_2_Sb_2_Te_5_ by single or multiple fs optical pulses. Here the dependence of the R and AR signals and the associated COP upon pump fluence was investigated with pump and probe electric fields parallel to the [110] and [100] axes respectively. A measurement was made on as-deposited material at elevated pump fluence and then immediately repeated with a reduced fluence of 0.42 mJ/cm^2^. The power spectra calculated from the repeated scans on Ge_2_Sb_2_Te_5_/GaSb and GaSb are shown in Fig. 4(a) and (b) respectively. The scans performed at the elevated fluence (not shown) exhibited similar behavior for both Ge_2_Sb_2_Te_5_/GaSb and GaSb. For small fluences the amplitudes of the R and AR signals increased linearly with pump fluence. COPs were clearly observed within the AR signal. While their amplitude increased linearly with pump fluence, their frequency and relaxation time were observed to decrease. For Ge_2_Sb_2_Te_5_/GaSb the relaxation time of the 3.4 THz mode decreased linearly from 660 fs to 470 fs as the fluence increased from 0.42 to 1.27 mJ/cm^2^, while for GaSb the relaxation time of the 6.77 THz mode decreased from 5.0 ps to 3.5 ps as the fluence increased from 0.42 to 1.70 mJ/cm^2^. Further details are provided in supplementary information S8.

The repeated scans at 0.42 mJ/cm^2^ fluence show that the 3.4 THz mode in Ge_2_Sb_2_Te_5_ vanished after exposure to a fluence of 1.70 mJ/cm^2^, while the 6.77 THz mode vanished after exposure to a fluence of 2.12 mJ/cm^2^ in both samples. COPs instead appeared in the AR signal at frequencies of 4.2 THz in both samples and at 3.1 THz for the Ge_2_Sb_2_Te_5_/GaSb sample. The 4.2 THz mode also appeared with large amplitude in the transient R signal (not shown). The 4.2 and 3.1 THz COPs may be attributed to the A_1g_ and E_g_ modes of elemental Sb, that segregates at the elevated temperatures induced by the pump^7^. The frequencies observed in the present study are about 0.4 THz lower and hence distinct from those commonly observed by others in the R signal from amorphous and polycrystalline Ge_2_Sb_2_Te_5_^21^,^36^.

In summary, comprehensive time-resolved reflectance and anisotropic reflectance measurements have been performed upon epitaxial Ge_2_Sb_2_Te_5_/GaSb(001) and GaSb(001) samples. The orientation of pump and probe electric fields have been varied relative to the crystallographic axes. The AR measurements are richly featured and yield relaxation times that can be ascribed to the interaction of the different populations of electrons and phonons. GaSb(001) is an exemplar in which the observation of the T_2_ transverse optical phonon has been shown to result from a combination of ISRS and the action of a SC field. The results can be fully understood both in terms of a theory based upon use of the Raman tensor, and also from a microscopic model of selective optical bond breaking. Epitaxial Ge_2_Sb_2_Te_5_/GaSb(100) exhibits both the T_2_ mode supported by the substrate and also a T_2_-like mode associated with the Ge_2_Sb_2_Te_5_. The insensitivity to the pump polarization suggests that the T_2_-like mode is excited by a SC field, either in the Ge_2_Sb_2_Te_5_ if the distorted rock salt structure lacks inversion symmetry, or by a SC field in the GaSb leading to impulsive excitation of the Ge_2_Sb_2_Te_5_ at the Ge_2_Sb_2_Te_5_/GaSb interface. The observation of the T_2_-like mode provides confirmation that the Ge_2_Sb_2_Te_5_ is essentially cubic and challenges mode assignments made in previous studies. The SC field also generates lateral stress in the lattice that leads to a long-lived transient AR response. This may be important for the generation of surface acoustic waves (SAWs) that can be steered by means of the optical polarization, without the need for an overlaid grating. Finally, the formation of a new phase after exposure to large pump fluence was observed in both Ge_2_Sb_2_Te_5_ and Ge_2_Sb_2_Te_5_/GaSb. The observed frequencies suggest segregation of Sb, while increased disorder in the GaSb and Ge_2_Sb_2_Te_5_ leads to the disappearance of the T_2_ and T_2_-like modes from the AR signal.

## Methods

Time-resolved R and AR (rotation) measurements were performed upon a Ge_2_Sb_2_Te_5_(15 nm)/GaSb(50–100 nm)/GaSb(001) structure prepared by molecular beam epitaxy. The Ge_2_Sb_2_Te_5_ exhibits a predominant cube on cube growth with the Ge_2_Sb_2_Te_5_ [100] axis parallel to the GaSb[100] axis^10^. Additional measurements were performed upon a reference structure without the Ge_2_Sb_2_Te_5_ layer. Pump and probe pulses of 800 nm wavelength and 55 fs duration were generated by a Ti:sapphire regenerative amplifier system at 100 kHz repetition rate. The linearly polarized pump pulse with fluence of 0.85 mJ/cm^2^ was directed onto the sample at close to normal incidence. The R and AR response were measured by a time delayed *s*-polarized probe pulse with fluence of 0.2 mJ/cm^2^ incident at 45° to the sample normal. An optical chopper was placed in the pump beam so that phase sensitive detection could be used to extract the transient R and AR signals (see Supplementary Information: S9). The polarization of the pump and probe beams relative to the crystallographic axes of the samples was controlled by rotating the sample about its normal, and by rotating a polarizer placed in the path of the pump beam.

## Author Contributions

F.K. and W.B. prepared the epitaxial Ge_2_Sb_2_Te_5_(001) and GaSb(001) samples. A.S., U.A.S.A., Y.L. and R.J.H. developed the measurement procedure. A.S., U.A.S.A. and Y.L. made the pump-probe measurements, Y.L. performed the fitting of the data, and A.S. prepared the figures. R.J.H. developed the microscopic and Raman tensor based models. G.P.S., C.D.W. and R.J.H. developed the interpretation of the data. R.J.H. conceived the project, while Y.L., A.S. and R.J.H. together wrote the paper.

## Additional information

Further underlying research materials may be found at http://hdl.handle.net/10871/13760

## Supplementary Material

Supplementary Information Observation of T2-like coherent optical phonons in epitaxial Ge2Sb2Te5/GaSb(001) films
