# Supplementary material for: Observation of T2-like coherent optical phonons in epitaxial Ge2Sb2Te5/GaSb(001) films
Source: Sci Rep. 2013 Oct 16;3:2965. doi: 10.1038/srep02965 (PMC3797426; doi:10.1038/srep02965)
Supplement: Supplementary Information — Observation of T2-like coherent optical phonons in epitaxial Ge2Sb2Te5/GaSb(001) films [file srep02965-s1.pdf]

Observation of T_2-like coherent optical phonons in epitaxial $Ge_2Sb_2Te_5$/GaSb(001) films

A. Shalini, Y. Liu, U. Al-Jarah, G. P. Srivastava, C. D. Wright, F. Katmis, W. Braun, and R. J. Hicken

Supplementary Information

In the following supplementary information we present further details of experimental and theoretical methods, a simple microscopic model of the response of GaSb to the polarized optical pump, and additional experimental results.

S1. Time resolved Reflectance and Anisotropic Reflectance of GaSb(001)

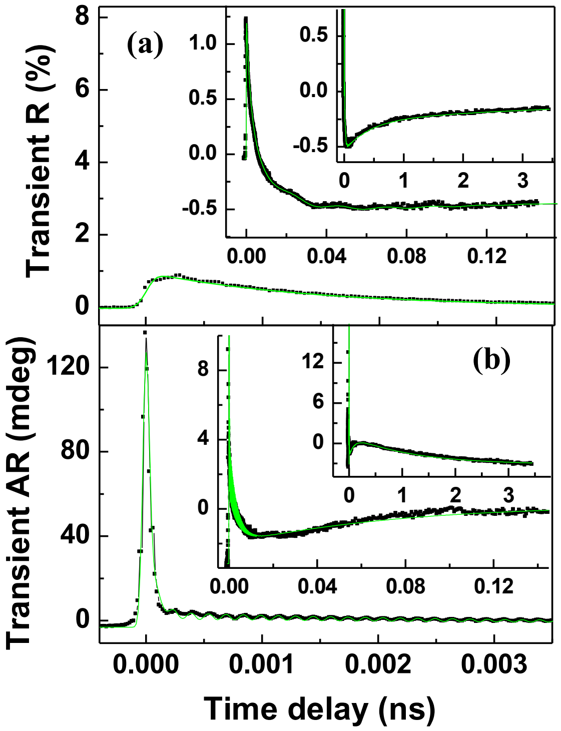

Fig S1.1: **Reflectance and Anisotropic Reflectance signal of GaSb: (a)** A typical time resolved reflectance signal obtained from the GaSb(001) substrate with the pump and probe electric fields parallel to the [110] and [100] axes respectively. The insets allow all the principal features of the signal to be seen clearly. The solid symbols represent the experimental data whereas the green lines are a fit to the function described in section S2. (b) A typical time resolved rotation signal obtained from the GaSb(001) substrate with the pump and probe electric fields parallel to the [110] and [100] axes respectively.

S2. Fitting time resolved Reflectance and Anisotropic Reflectance measurements

The reflectance (R) and anisotropic reflectance (AR) were fitted to the general form

$$\begin{aligned} &A\exp\left[-\frac{(t-t_0)^2}{4w^2}\right]+\sum_i\frac{B_i}{2}\exp\left[\frac{w^2}{\tau_i^2}-\frac{(t-t_0)}{\tau_i}\right]\left\{1-erf\left[\frac{w}{\tau_i}-\frac{(t-t_0)}{2w}\right]\right\} \\ &+\sum_j\frac{C_j}{2}\exp\left[-\frac{(t-t_0)}{\tau_j}\right]\left\{1-erf\left[-\frac{(t-t_0)}{2w}\right]\right\}\cos\left[\omega_j(t-t_0)+2\pi\beta_j(t-t_0)^2+\phi_j\right] \end{aligned}, \text{(SE1)}$$

in which t and t_0 are the times of arrival of the probe and pump respectively. The first term with amplitude A describes an initial Gaussian peak with $1/e^2$ full width of $2\sqrt{2}w$. A series of Gaussian error functions with amplitudes B_i, in which a simple exponential relaxation is convolved with a Gaussian impulse, is used to describe the multi-exponential background within the signal. Finally, a series of terms with amplitude C_j represent the damped oscillatory response of the phonon modes to a Gaussian impulse. The cosine factor oscillates with circular frequency ω_j, has initial phase ϕ_j, and includes a term with coefficient $2\pi\beta_j$ that describes any chirp of the phonon associated with the transient temperature profile. A constant offset was also included to account for the time independent finite signal sometimes observed at negative time delay which can arise from diffuse scatter of the pump beam from the sample surface into the detector.

Table S2.1 shows fitted parameter values for the data obtained from the $Ge_2Sb_2Te_5$/GaSb(001) sample shown in Figure 1 of the main paper, and for a comparable set of data (shown in Supplementary Information S1) obtained from the GaSb(001) sample. The pump and probe electric fields were parallel to the [110] and [100] axes of the sample respectively. The Gaussian peak with amplitude A and 4 relaxation terms with coefficients B_i were used to fit each of the signals. No attempt was made to fit the weak 4.2 – 4.5 THz oscillations observed in the R signal from the GaSb/ $Ge_2Sb_2Te_5$ sample. Instead a single oscillatory term was included so as to fit the low frequency oscillations observed in both samples. For the AR signals, one and two oscillatory components were required to fit the data from the GaSb and $Ge_2Sb_2Te_5$/GaSb samples respectively.

The fitted parameter values for the R signals are broadly similar for the two samples The Gaussian peak is small in comparison to the other terms and so the difference in the fitted values of A is not significant. For the AR signals, the relaxation times τ_2, τ_3 and τ_4 associated with the terms with coefficients B_2, B_3, and B_4 are also similar for the two samples although B_2 and B_3 have different signs. The relaxation times for R and AR signals are seen to be quite different, suggesting that the underlying physical processes are different.

In the case of the AR signals, the non-oscillatory components of the fitting function were subtracted from the experimental data to leave only an oscillatory residual. The residual was then fitted by the damped oscillatory function described within the main text. The fitted values of the frequency, relaxation time and initial phase were found to be almost identical to those obtained from the fitting of equation (1).

				t_0, τ_i	ω_i	φ_i	w	offset
**Rotation**	**$Ge_2Sb_2Te_5$/ GaSb**	A	98.91	5.0 fs	–	–	–	–
		B_1	19.23	500 fs	–	–	–	–
		B_2	-28.99	3.37 ps	–	–	–	–
		B_3	6.64	137 ps	–	–	–	–
		B_4	11.93	1.6 ns	–	–	–	–
		C_1	14.42	430 fs	3.40 THz	0.316	–	–
		C_2	1.46	2.98 ps	6.77 THz	1.530	30 fs	-0.290
	**GaSb**	A	97.79	0.8 fs	–	–	–	–
		B_1	48.37	45 fs	–	–	–	–
		B_2	2.32	3.24 ps	–	–	–	–
		B_3	-1.57	106 ps	–	–	–	–
		B_4	2.19	1.14 ns	–	–	–	–
		C_1	0.84	3 ps	6.77 THz	2.540	20 fs	-3.150
		C_2	0	–	–	–	–	–
**Reflect-ance**	**$Ge_2Sb_2Te_5$ /GaSb**	A	0.00016	6 fs	–	–	–	–
		B_1	0.97	1.44 ps	–	–	–	–
		B_2	0.43	23.6 ps	–	–	–	–
		B_3	-0.21	0.38 ns	–	–	–	–
		B_4	-0.18	4.3 ns	–	–	–	–
		C_1	0.078	23 ps	46 GHz	-1.75	50 fs	-0.03
		C_2	0	–	–	–	–	–
	**GaSb**	A	-0.016	5 fs	–	–	–	–
		B_1	0.49	2.04 ps	–	–	–	–
		B_2	0.39	13.1 ps	–	–	–	–
		B_3	-0.14	0.424 ns	–	–	–	–
		B_4	-0.11	5.4 ns	–	–	–	–
		C_1	-0.017	50 ps	43 GHz	-3.23	40 fs	-0.04
		C_2	0	–	–	–	–	–

Table S2.1: Parameter values obtained by fitting equation SE1 to example R and AR signals from the GaSb and GaSb/ $Ge_2Sb_2Te_5$ samples.

S3. Dependence of slow transient Anisotropic Reflectance signal acquired from GaSb(001) upon pump and probe polarization

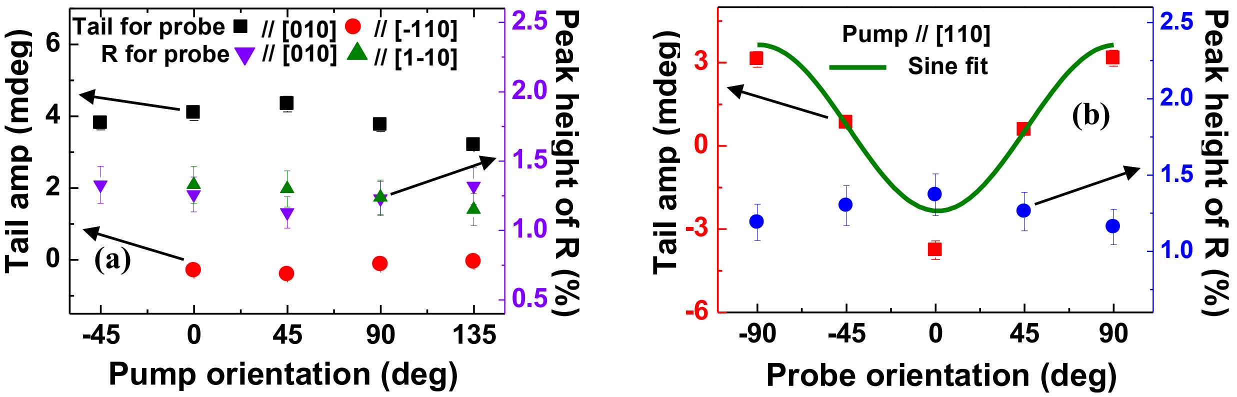

Fig S3.1: Dependence of the amplitude B_4 of the slow transient observed within the AR signal of GaSb(001) upon the orientation of (a) the pump and (b) the probe electric field. The peak R signal value is shown for comparison.

S4. Theory of Transient Stimulated Raman Scattering

In the theory described by Merlin[11], transient stimulated Raman scattering is considered as a two-stage process. The excitation of a coherent optical phonon (COP) with amplitude $Q(\mathbf{r},t)$ by the pump electric field **E**, through the process of conventional impulsive stimulated Raman scattering (ISRS) is described by the equation of motion

$$\ddot{Q}(\mathbf{r},t)+\Gamma\dot{Q}(\mathbf{r},t)+\frac{1}{8\pi^3}\int\Omega_{\mathbf{q}}^2 e^{-i\mathbf{q}\cdot(\mathbf{r}-\mathbf{r}')}Q(\mathbf{r}',t)d^3r'd^3q=\frac{1}{2}\sum_{kl}\left(\frac{\partial\chi_{kl}^i}{\partial Q}Q_iE_kE_l\right) \text{(SE2)}$$

in which **q** is the phonon wavevector, $\Omega_{\mathbf{q}}$ is the phonon frequency, and Γ is the phonon damping parameter. The driving force is seen to contain the 2nd rank Raman tensor $R=\frac{\partial\chi_{kl}^i}{\partial Q}Q_i$. The solution of (SE2) yields

$$Q\propto\sum_{mp}\left(\frac{\partial\chi_{mp}}{\partial Q}\cos(\alpha_m)\cos(\alpha_p)\right) \text{(SE3)}$$

in which $\cos(\alpha_i)$ are the direction cosines of the pump electric field. Alternatively, if the phonon is instead excited by an optically generated space charge (SC) field, then it is the SC field that appears on the right hand side of (SE2) and Q is independent of the pump beam polarization.

By solving the wave equation for the probe electric field in the sample medium, with the electric susceptibility modified by the presence of the phonon, the transient reflectance $\Delta R/R$ detected by the probe is found to be proportional to the phonon amplitude Q and an additional factor

$$\sum_{kl}\left(\frac{\partial\chi_{kl}}{\partial Q}\cos(\beta_k)\cos(\beta_l)\right) \text{(SE4)}$$

in which $\cos(\beta_i)$ are the direction cosines of the probe beam. If the phonon is excited by a combination of ISRS and the action of a SC field then

$$\Delta R/R\sim\left[C+\sum_{mp}\left(\frac{\partial\chi_{mp}}{\partial Q}\cos(\alpha_m)\cos(\alpha_p)\right)\right]\sum_{kl}\left(\frac{\partial\chi_{kl}}{\partial Q}\cos(\beta_k)\cos(\beta_l)\right) \text{(SE5)}$$

where the constant C represents the effect of the SC field and must be complex to describe both the amplitude and phase of the transient reflectance signal.

Next it is necessary to consider the measurement geometry and operation of the detector. While the pump beam is incident at near normal incidence, it is convenient to use a larger angle of incidence for the probe beam so that the beams are spatially separated and can be controlled independently. However strong refraction occurs at the air-sample interface (the refractive indices of GaSb and $Ge_2Sb_2Te_5$ are 4.4+0.38i [ref www.luxpop.com] and 4.6+4.3i [K. Shportko et al. Nat. Mat. **7**, 653 (2008)] respectively for 800 nm wavelength) so that both beams can be considered as

propagating roughly parallel to the [001] axis within the medium. In addition s-polarization is chosen for the probe. Therefore both pump and probe electric fields can be assumed to lie within the (001) plane parallel to the $(\cos\varphi, \sin\varphi, 0)$ and $(\cos\theta, \sin\theta, 0)$ directions assuming the coordinate system of Figure 2(b).

The polarizing bridge detector is balanced for the DC probe signal. Therefore the beams entering the two photodiode detectors within the bridge have their electric field parallel to the $(\cos(\theta+\pi/4), \sin(\theta+\pi/4), 0)$ and $(\cos(\theta-\pi/4), \sin(\theta-\pi/4), 0)$ directions. The transient reflectance $(\Delta R/R)_i$ from the two diodes may then be written as

$$(\Delta R/R)_1 \propto (\cos(\theta+\pi/4), \sin(\theta+\pi/4), 0)\,\mathrm{M}\begin{pmatrix} \cos(\theta+\pi/4) \\ \sin(\theta+\pi/4) \\ 0 \end{pmatrix} \quad \text{(SE6)}$$

$$(\Delta R/R)_2 \propto (\cos(\theta-\pi/4), \sin(\theta-\pi/4), 0)\,\mathrm{M}\begin{pmatrix} \cos(\theta-\pi/4) \\ \sin(\theta-\pi/4) \\ 0 \end{pmatrix} \quad \text{(SE7)}$$

in which M is the matrix representing the Raman tensor for the particular phonon mode within the coordinate system defined by the crystallographic axes of the sample. The detector electronics outputs signals

$$S_R = (\Delta R/R)_1 + (\Delta R/R)_2, \quad \text{and} \quad S_{AR} = (\Delta R/R)_1 - (\Delta R/R)_2, \quad \text{(SE8)}$$

which correspond to the transient reflectance and rotation respectively.

The T_d and O_h space groups have 5 irreducible representations A_1, A_2, E, T_1 and T_2 with the following matrix representations of the Raman tensor

$$A \begin{pmatrix} a & 0 & 0 \\ 0 & a & 0 \\ 0 & 0 & a \end{pmatrix}; \quad E \begin{pmatrix} b & 0 & 0 \\ 0 & b & 0 \\ 0 & 0 & b \end{pmatrix};$$

$$T_x \begin{pmatrix} 0 & 0 & 0 \\ 0 & 0 & d \\ 0 & d & 0 \end{pmatrix}; \quad T_y \begin{pmatrix} 0 & 0 & d \\ 0 & 0 & 0 \\ d & 0 & 0 \end{pmatrix}; \quad T_z \begin{pmatrix} 0 & d & 0 \\ d & 0 & 0 \\ 0 & 0 & 0 \end{pmatrix}. \quad \text{(SE9)}$$

Finally it remains only to combine equations (SE5) – (SE9) and calculate the R and AR signal for each mode. For the A and E modes, S_R has constant values proportional to a and b respectively, while S_{AR} vanishes. For T_x and T_y both S_R and S_{AR} vanish, but for T_z, S_R vanishes while

$$S_{AR} \propto (C + \sin 2\varphi)\cos 2\theta. \quad \text{(SE10)}$$

The form of (SE10) fully describes the experimental results presented in Fig 2.

S5. A microscopic model for the response of GaSb(001)

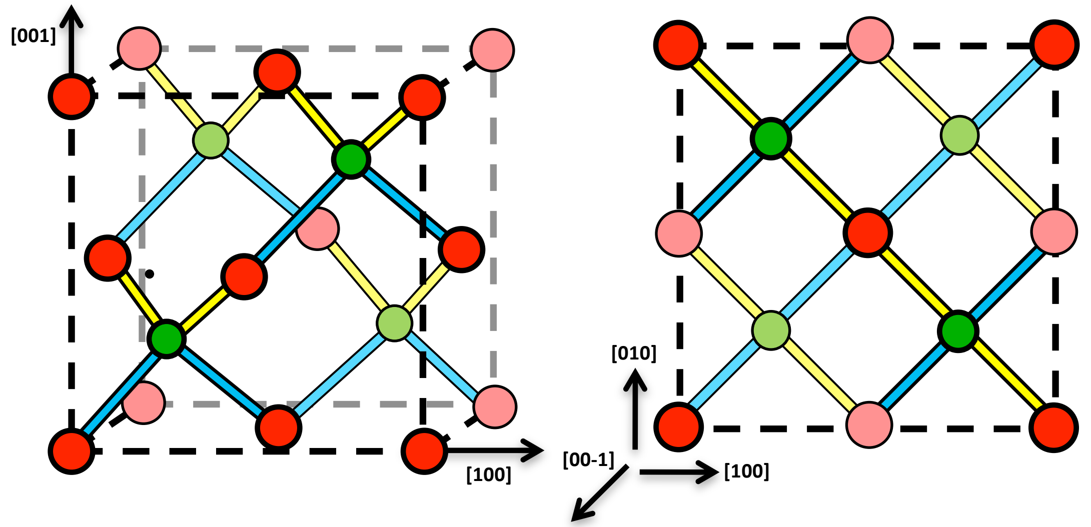

Fig. S5.1: Zincblende crystal structure of GaSb in (a) 3 dimensions and (b) viewed along the [001] axis. The green and red spheres indicate the 2 sub-lattices, while inequivalent bonds are shaded in yellow and blue.

The zincblende crystal structure of GaSb is shown in Fig S5.1. GaSb lacks inversion symmetry, and is both polar and piezoelectric. While all atoms have tetrahedral coordination, the yellow and blue bonds lie in orthogonal planes and are inequivalent. When the electric field of the pump lies along the [110] direction, it describes angles of 35^0 and 90^0 with the blue and yellow bonds respectively. Assuming that the excitation of the bond varies as the scalar product of the electric field and a unit vector parallel to the bond, then the blue bonds are excited more strongly. The Ga and Sb atoms experience unequal force from the blue and yellow bonds, causing them to move in opposite directions parallel to the [001] axis. This is exactly the displacement of basis atoms associated with the T_{2z} optical phonon. If the pump electric field instead lies parallel to the $\left[1\bar{1}0\right]$ direction then the role of the blue and yellow bonds is reversed and the direction of displacement of the two basis atoms is interchanged. The phase of the phonon excited by the pump will therefore change by π radians as the pump electric field is rotated from [110] to $\left[1\bar{1}0\right]$[23]. The optically induced difference in bond strength is short-lived. However once the T_{2z} mode has been excited, the oscillatory displacement of the basis atoms causes the yellow and blue bonds to be alternately stretched and compressed. This leads to an optical birefringence that produces maximum (zero) optical rotation when the probe electric field lies along the <100> (<110>) axes. The generation (and detection) of the COP observed in the AR data is hence understood in terms of selective bond breaking.

Consider the green atom with coordinates (1/4,1/4,1/4) within the conventional unit cell shown in Fig 1(a). The red nearest neighbours at A (0,0,0), B (1/2,1/2,0), C (1/2,0,1/2) and D (0,1/2,1/2) give rise to bonds that lie parallel to the $\left[\bar{1}\bar{1}\bar{1}\right]$, $\left[11\bar{1}\right]$, $\left[1\bar{1}1\right]$ and $\left[\bar{1}11\right]$ directions respectively. Let us assume that the extent of excitation of each type of bond (i.e. the number of bonding to anti-bonding transitions that occur) is proportional to $\left(\mathbf{E}\cdot\hat{\mathbf{u}}\right)^2$, where $\mathbf{E}$ is the electric field of the pump beam and

$\hat{\mathbf{u}}$ is a unit vector parallel to the length of the bond, as expected for an electric dipole transition. If $\mathbf{E} = E_{0,pump}(\cos\varphi, \sin\varphi, 0)$, then the net force on the green atom is proportional to

$$(\mathbf{E}\cdot\hat{\mathbf{u}})_A^2 + (\mathbf{E}\cdot\hat{\mathbf{u}})_B^2 - (\mathbf{E}\cdot\hat{\mathbf{u}})_C^2 - (\mathbf{E}\cdot\hat{\mathbf{u}})_D^2 = 4E_{0,pump}^2 \sin(2\varphi) \quad \text{(SE11)}$$

The difference in bond strength and orientation associated with the optical pumping and the displacement of the atoms leads to inequivalent reflection coefficients of $(r + \delta r/2)$ and $(r - \delta r/2)$ when the electric field of the probe beam is polarized parallel to the $[110]$ and $[\bar{1}10]$ directions respectively. If the incident probe electric field has the form $\mathbf{E} = E_{0,probe}(\cos\theta, \sin\theta, 0)$ then the AR signal (S_{AR}) is equal to the difference in reflected intensity of incident beams with electric field $\mathbf{E}_1 = \frac{E_{0,probe}}{\sqrt{2}}\left(\cos\left(\theta + \frac{\pi}{4}\right), \sin\left(\theta + \frac{\pi}{4}\right), 0\right)$ and $\mathbf{E}_2 = \frac{E_{0,probe}}{\sqrt{2}}\left(\cos\left(\theta - \frac{\pi}{4}\right), \sin\left(\theta - \frac{\pi}{4}\right), 0\right)$.
The reflected electric field amplitudes may be written as

$$\mathbf{E}_1 = \frac{E_{0,probe}}{2}\begin{pmatrix} (r+\delta r/2)\left[\cos\left(\theta + \frac{\pi}{4}\right) + \sin\left(\theta + \frac{\pi}{4}\right)\right] \\ (r-\delta r/2)\left[-\cos\left(\theta + \frac{\pi}{4}\right) + \sin\left(\theta + \frac{\pi}{4}\right)\right] \\ 0 \end{pmatrix} = \frac{E_{0,probe}}{\sqrt{2}}\begin{pmatrix} (r+\delta r/2)\cos\theta \\ (r-\delta r/2)\sin\theta \\ 0 \end{pmatrix}$$

(SE12)

$$\mathbf{E}_1 = \frac{E_{0,probe}}{2}\begin{pmatrix} (r+\delta r/2)\left[\cos\left(\theta - \frac{\pi}{4}\right) + \sin\left(\theta - \frac{\pi}{4}\right)\right] \\ (r-\delta r/2)\left[-\cos\left(\theta - \frac{\pi}{4}\right) + \sin\left(\theta - \frac{\pi}{4}\right)\right] \\ 0 \end{pmatrix} = \frac{E_{0,probe}}{\sqrt{2}}\begin{pmatrix} (r+\delta r/2)\sin\theta \\ -(r-\delta r/2)\cos\theta \\ 0 \end{pmatrix}$$

so that

$$S_{AR} \propto E_1^2 - E_2^2 = \frac{E_{0,probe}^2}{2}\left\{\begin{matrix} \left[(r+\delta r/2)\cos\theta\right]^2 + \left[(r-\delta r/2)\sin\theta\right]^2 \\ -\left[(r+\delta r/2)\sin\theta\right]^2 - \left[-(r-\delta r/2)\cos\theta\right]^2 \end{matrix}\right\}$$
$$= E_{0,probe}^2 \cos(2\theta)\, r\,\delta r \quad \text{(SE13)}$$

Since $\delta r \propto \sin(2\varphi)$, we hence find that $S_{AR} \propto \sin(2\varphi)\cos(2\theta)$. Hence the simple model yields the same dependence of the AR signal upon the pump and probe polarization as predicted by the Raman tensor description of ISRS presented in section S4.

This simple model can also be extended to describe the effect of a SC field parallel to the [001] axis that occurs due to the optical excitation of electron-hole pairs and their separation within the built-in potential near the interface. The SC field again causes Ga and Sb ions to be accelerated in opposite directions. The initial impulse associated with the rise of the SC field excites the T_{2z} mode. However the slow relaxation of the SC field causes a long-lived non-oscillatory displacement of the basis ions and an inequivalence of the blue and yellow bonds. This is the origin of the slow transient rotation observed when the probe electric field lies parallel to a <100> axis. The generation of the SC field is independent of pump polarization, while the amplitude of the slow transient again has a $\cos(2\theta)$ dependence on the probe polarization.

S6. Fitting the dependence of the phonon amplitude and phase upon the pump polarization

ISRS and a SC field are expected to excite COPs of different amplitude and phase. For the specific case of a GaSb(001) surface, the pump and probe electric fields are assumed to lie in the plane of the surface and describe angles φ and θ respectively with the [100] axis. When the two mechanisms act simultaneously, the displacement of an ion associated with the resulting COP, and also the time resolved anisotropic reflectance (AR), may be represented by an oscillatory function of the form

$$\mathrm{Re}\left[Ae^{(i\omega t+\varepsilon)}\right]=\cos(2\theta)\mathrm{Re}\left[A_1\sin(2\varphi)e^{(i\omega t+\varepsilon_1)}+A_2e^{(i\omega t+\varepsilon_2)}\right] \quad \text{(SE14)}$$

where A_1 and ε_1 are the amplitude and phase associated with ISRS, A_2, and ε_2 are associated with the SC field, and A and ε are the net amplitude and phase.

The signal amplitude is maximum when the probe electric field is applied parallel to a <100> axis. If the pump polarization is then varied, the complex signal $z = Ae^{i\varepsilon}$ traces out a straight line segment in the Argand plane of form

$$z = z_0 + \lambda(z_1 - z_0) = Ae^{i\varepsilon} \quad \text{(SE15)}$$

with end points corresponding to $\lambda = \sin(2\varphi) = +/-1$. The values of z_0 and z_1 may be obtained immediately by fitting a straight line to the data within the Argand plane as shown in Fig. 2(f). The dependence of the amplitude and phase of the AR signal upon the pump polarization then follows immediately. The square of the amplitude

$$A^2 = \{z_0{}^* + \lambda(z_1{}^* - z_0{}^*)\}\{z_0 + \lambda(z_1 - z_0)\} = P + Q\sin 2\varphi + R\sin^2 2\varphi \quad \text{(SE16)}$$

where

$$P = x_0^2 + y_0^2;\ Q = [x_0(x_1 - x_0) + y_0(y_1 - y_0)];\ R = [(x_1 - x_0)^2 + (y_1 - y_0)^2] \quad \text{(SE17)}$$

The tangent of the phase

$$\tan\varepsilon = \frac{Im\,(z)}{Re\,(z)} = \frac{y_0 + H\sin 2\varphi}{x_0 + K\sin 2\varphi}, \quad \text{(SE18)}$$

where $H = y_1 - y_0$ and $K = x_1 - x_0$. The expressions in (SE16) – (SE18) are then used to generate the fit curves of the dependence of phase and amplitude upon pump polarization in Fig 2(d) and 2(e) respectively.

S7. Additional pump-probe data obtained from $Ge_2Sb_2Te_5$/GaSb(001)

Two phonon modes with frequencies of about 3.4 THz and 6.8 THz were observed within the AR signal from the $Ge_2Sb_2Te_5$/GaSb(001) sample, there being some small variation in the precise values obtained from the full set of measurments. The main text describes how the amplitude and phase of the 3.4 THz phonon varied with the polarization of the pump and probe. Measurements were made in two sets where firstly, only the pump polarization was changed, and secondly, where the pump polarization and sample orientation were changed together so that effectively the probe electric field was being rotated relative to the electric field of the pump and the crystallographic axes of the sample. Further data obtained by means of these methods is shown in in Figure S7.1. From panels (a)-(d) the frequency of both phonon modes is seen to be essentially independent of pump and probe polarization within experimental error. From Fig 1(a) the initial phase of the 3.4 THz mode is seen to vary by only 0.7 rads, and has no clear dependence upon pump polarization, when the probe electric field was parallel to the [010] axis. In Fig 1(a) the initial phase of the 3.4 THz phonon oscillation appears to roughly alternate between two values different by π rads when the pump polarization is kept fixed, in agreement with the models of sections S5 and S6. Panels (c) and (d) also show the pump and probe polarization dependence of the amplitude of the 6.8 THz mode. The dependence upon pump and probe polarization is similar to that observed for GaSb(001) and hence in full agreement with the models of section S4 and S5. Since the amplitude of the 6.8 THz mode was an order of magnitude smaller than that of the 3.4 THz mode, there is greater uncertainty in the extracted amplitude of the 6.8 THz mode, while its initial phase could not be extracted reliably and so has not been presented. The dependence of the amplitude of the slow transient response (the quantity B_4 within section S2) on pump and probe orientation is displayed in Fig S7.1 (e) and (f) respectively, and is similar to that observed for the GaSb(001) sample. Interestingly the amplitude of this slow transient is some 3 times larger for the $Ge_2Sb_2Te_5$/GaSb(001) sample.

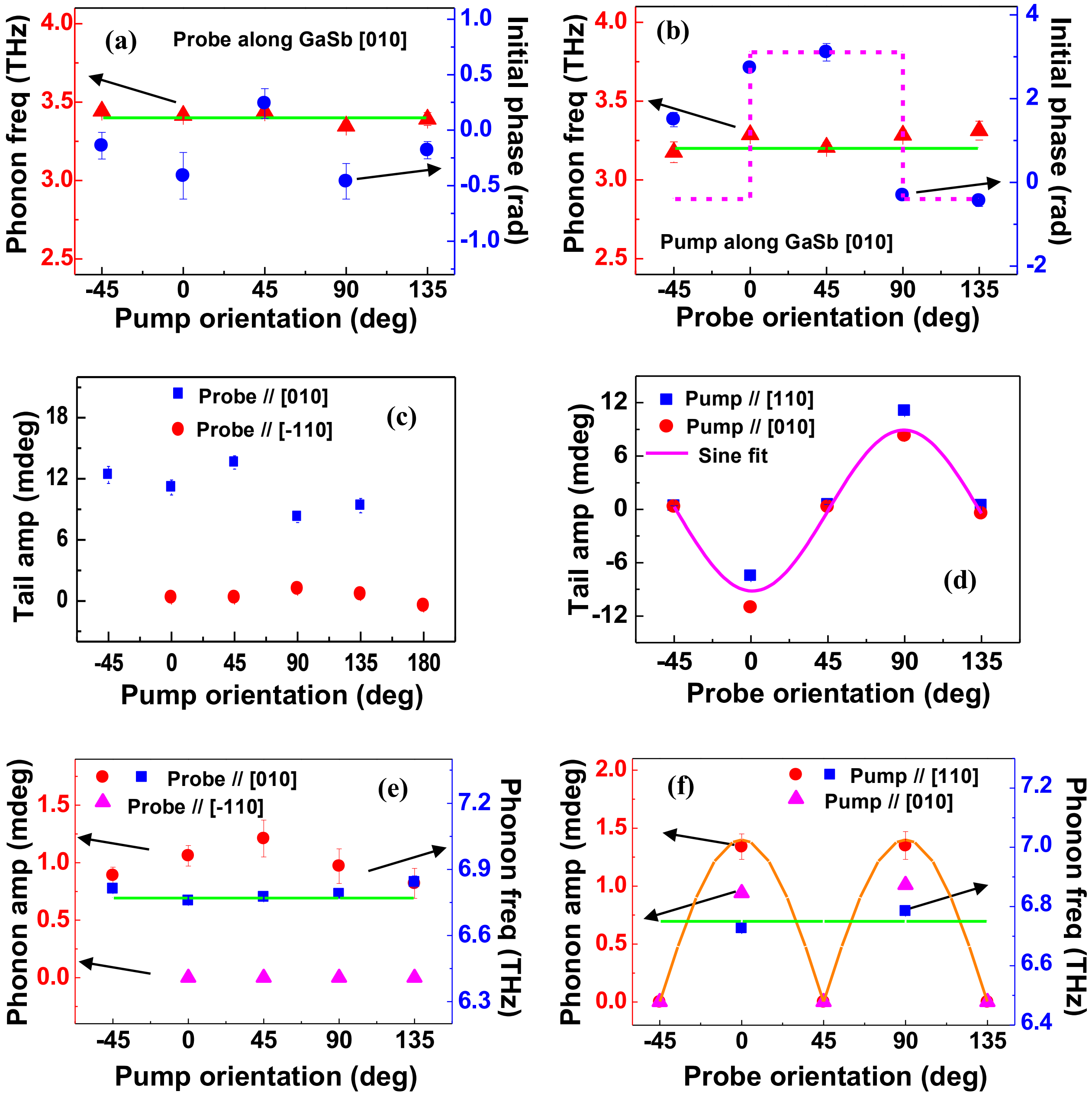

Fig S7.1 Variation of features of the AR signal from the $Ge_2Sb_2Te_5$/GaSb(001) sample as either the pump polarization was varied, in (a), (c) and (e), or the pump polarization and sample were rotated together so that the probe electric field was effectively rotated, in (b), (d) and (e). (a) and (b) show the dependence of the frequency and phase of the lower frequency phonon mode on pump and probe polarization respectively. (c) and (d) show the dependence of the amplitude of the slow transient response (tail) on pump and probe polarization. (e) and (f) show the dependence of amplitude and frequency of the higher frequency mode upon pump and probe polarization.

S8. The effect of elevated pump fluences.

Further details of the effect of elevated pump fluences upon R and AR signals obtained from $Ge_2Sb_2Te_5$/GaSb(001) and GaSb(001) are presented in this section. The pump and probe electric fields were parallel to the [110] and [100] axes respectively in all measurements. Measurements were first performed on the sample in the as-deposited state with an elevated pump fluence and then immediately repeated at a lower pump fluence.

Fig S8.1 (a) and (b), respectively show the transient AR and R signals obtained from GaSb(001) with an elevated pump fluence. The oscillatory components of the transient AR and R signals obtained from the repeated measurement at a reduced fluence of 0.42 mJ/cm^2 are shown in (c) and (d), while the corresponding power spectra are shown in panels (e) and (f). The amplitude of the oscillatory component (not shown) measured at elevated fluences up to 1.70 mJ/cm^2 was found to have a sub-linear dependence on fluence as shown in panel (g). The frequency and the relaxation time (parameter τ_2 as defined in section S2) of the oscillations are also shown in panel (g) and both decrease with increasing pump fluence. This result is consistent with theoretical calculations that will be presented elsewhere. The hot zone-centre phonons excited by the optical pump can decay into two (or more) phonon modes via a combination of the (dominant) Klemens channel Γ(TO) -> TA + LA and the (weak) Ridley channel Γ(TO) -> TA + LO.

The repeated measurements at lower pump fluence show that the GaSb(001) undergoes a structural phase transition when the pump fluence exceeds 1.70 mJ/cm^2. The amplitude of the T_2 optical phonon is decreased while two new strong coherent phonon modes appear in the AR signal with frequencies of 4.23 and 3.06 THz. The phonon with frequency of 4.23 THz also appears in the R signal. The relaxation times for these two phonons were found to be about 1.0 ps, which is significantly shorter than that of the orignial T_2 phonon. The new phonons may be respectively attributed to the A_{1g} and E_g modes of Sb, which may segregate from the GaSb when high pump fluences are applied. However, the frequencies observed in the present study are about 0.4 THz lower than those observed by other groups.[21,36] Finally, the peak height of the AR and R signals obtained from the measurements at elevated fluence are plotted in Fig 9.1(h) and are seen to exhibit a roughly linear dependence upon the pump fluence.

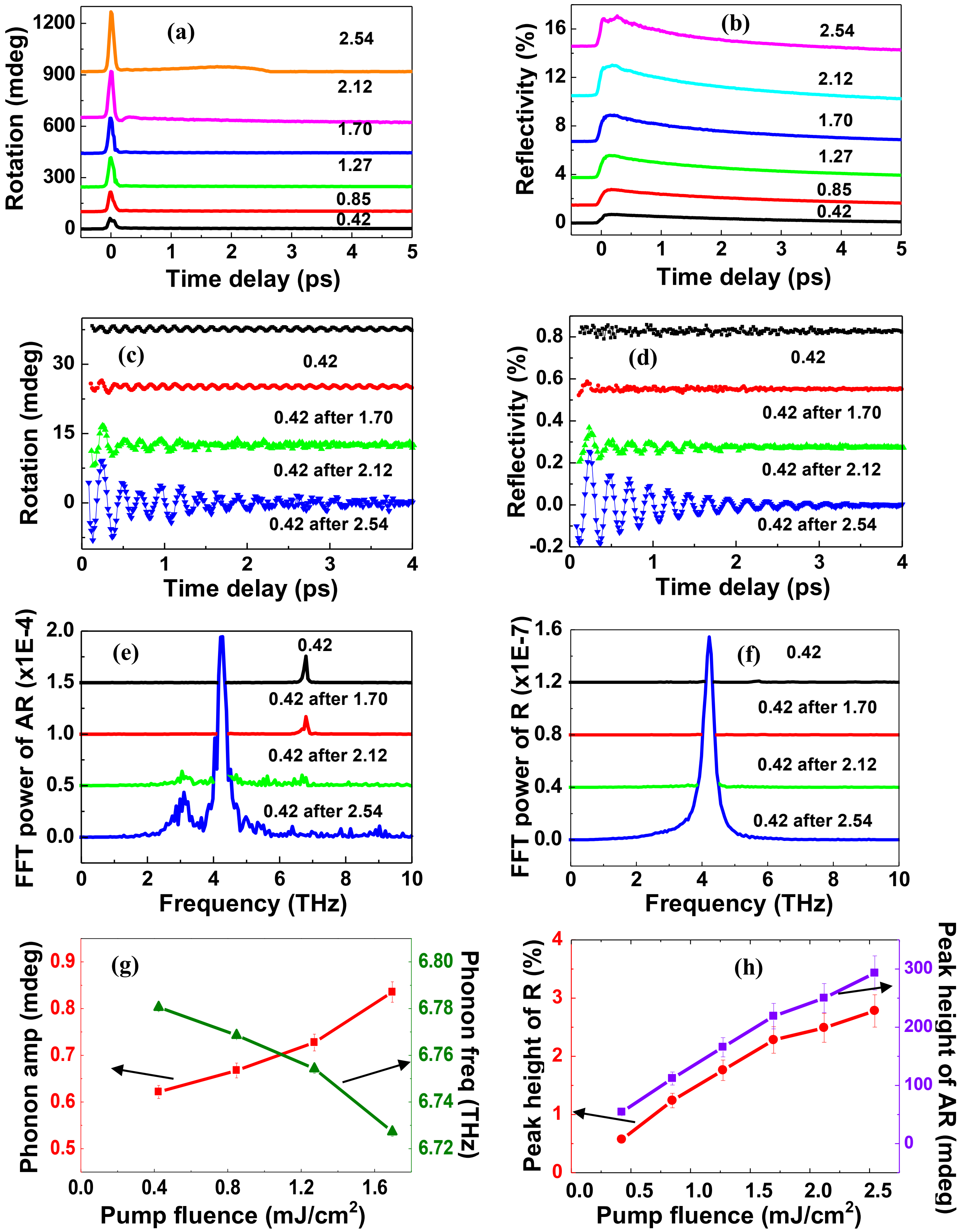

Fig S8.1 Effect of elevated pump fluence upon the transient R and AR signals obtained from GaSb(001). (a) and (b) transient AR and R signals respectively, obtained at the values of the elevated fluence indicated in the figure. (c) and (d) oscillatory components of the AR and R signals respectively, obtained with 0.42 mJ/cm^2 pump fluence after exposure to higher pump fluence. (e) and (f) FFT power spectra of the AR and R signals in (c) and (d) respectively. (g) dependence of the phonon amplitude, frequency and relaxation time during measurement at the elevated pump fluence. (h) height of the initial peaks in the R and AR signals in (a) and (b) are plotted against pump fluence.

The results of measurements made upon $Ge_2Sb_2Te_5$/GaSb(001) are shown in Fig S8.2. Panels (a) and (b) respectively show the transient R and AR data obtained at elevated fluence. The oscillatory components of the transient AR and R signals extracted from repeated scans at low fluence are shown in (c) and (d) while their respective power spectra are shown in (e) and (f). The peak heights of the AR and R signals in (a) and (b) are plotted in (g), and are seen to exhibit a linear dependence upon the pump fluence. Clear oscillatory components (not shown) with frequency of about 3.4 THz were observed in measurements made at elevated pump fluences in the range 0.42 to 1.06 mJ/cm^2. Panel (h) shows how the amplitude increased linearly above a threshold fluence of about 0.4 mJ/cm^2 and the frequency decreased linearly with increasing fluence. The phonon relaxation time (parameter τ_2 of section S2) was found to decrease from 660 fs at 0.42 mJ/cm^2 to 470 fs at 1.06 mJ/cm^2. The amplitude became suddenly weaker for higher fluences, suggesting that a structural change had occurred within the $Ge_2Sb_2Te_5$.

The repeated scans at low fluence showed that the phonon with frequency of about 3.4 THz disappeared after exposure to an elevated pump fluence greater than or equal to 1.27 mJ/cm^2, while the optical phonon with frequency of about 6.8 THz, supported by the homo-epitaxial GaSb underlayer, persisted until the pump fluence reached 2.12 mJ/cm^2. After exposure to higher fluences, the repeated scans show a new coherent phonon mode with frequency of 4.2 THz in both the AR and R signals. Since the new mode is observed in both $Ge_2Sb_2Te_5$/GaSb(001) and GaSb(001), it is thought to be the A_{1g} mode of crystalline Sb, which forms through segregation of Sb.

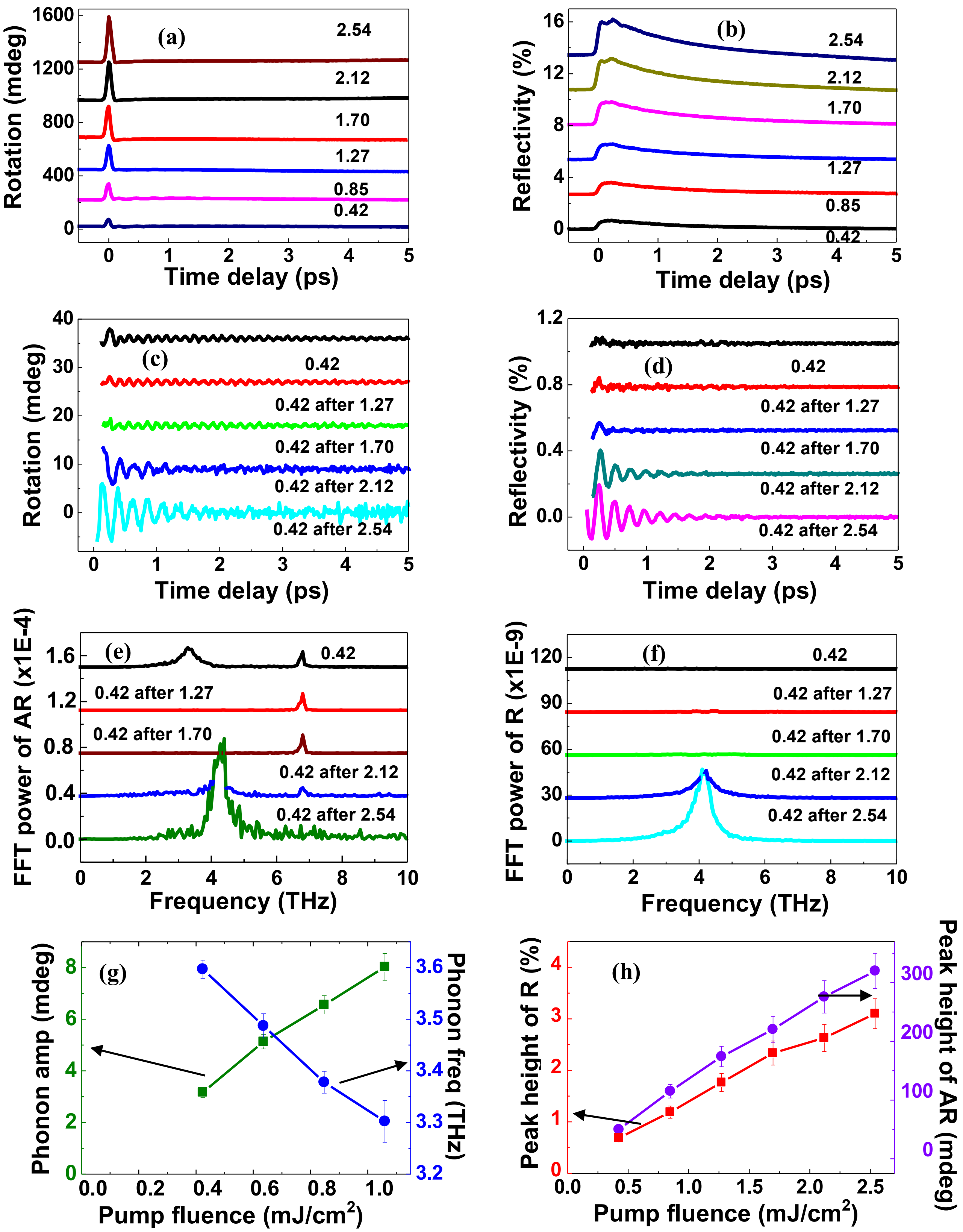

Fig S8.2 Effect of elevated pump fluence upon the transient R and AR signals obtained from $Ge_2Sb_2Te_5$/GaSb(001). (a) and (b) AR and R signals obtained at elevated pump fluence. (c) and (d) oscillatory AR and R signals obtained from repeated measurments at 0.42 mJ/cm^2 pump fluence. (e) and (f) FFT power spectra of the scans in (c) and (d) respectively. The variation of the phonon amplitude and frequency is shown in (g), while height of the initial peak in the R and AR signals from (a) and (b), is plotted against pump fluence in (h).

S9. Optical Pump Probe Method

Alignment of the optical measurement apparatus requires considerable care. A DataRay WinCamD-UCD12 beam profiler was used to characterize the size of the focused pump and probe spots and optimize their overlap. At normal incidence upon the beam profiler the intensity profiles of the spots along their principal axes were observed to be Gaussian with $1/e^2$ full widths of 100 μm × 120 μm and 30 μm × 40 μm for the pump and probe respectively. Overlap was performed with the beam profiler at the same position and orientation as the sample. The pulse duration was adjusted by using pre-compensation within the compressor stage of the regenerative amplifier system. The same setting gave the minimum pulse width for the pump and probe beams at the sample position, namely an intensity autocorrelation width of 80 ± 2 fs that corresponds to a bandwidth limited hyperbolic secant squared with full width half maximum of 55 fs. The pulses must be unchirped when they arrive at the sample or else strong variations of the AR signal may be induced close to the zero time delay condition. The reflected probe beam was incident upon a balanced polarization bridge detector comprised of a polarizing beam splitter and two photodiodes. The sum and difference of the photodiode outputs yield the R and AR signals respectively. Each signal was sent to a separate dual-phase lock-in amplifier so that the transient R and AR signals induced by the pump beam could be extracted. The probe beam polarization was set precisely to the s-state so as to avoid breakthrough of the transient R signal into the AR channel.
